# Increase in ECHOvirus 6 infections associated with neurological symptoms in the Netherlands, June to August 2016

**DOI:** 10.2807/1560-7917.ES.2016.21.39.30351

**Published:** 2016-09-29

**Authors:** Kimberley SM Benschop, Felix Geeraedts, Barbara Beuvink, Silke A Spit, Ewout B Fanoy, Eric CJ Claas, Suzan D Pas, Rob Schuurman, Jaco J Verweij, Sylvia M Bruisten, Katja C Wolthers, Hubert GM Niesters, Marion Koopmans, Erwin Duizer, on behalf of VIRO-TypeNed

**Affiliations:** 1 Center for Infectious Disease Control, National Institute for Public Health and the Environment, Bilthoven, the Netherlands; 2 Laboratory for Medical Microbiology and Public Health, Hengelo, the Netherlands; 3 Public Health Service, Twente, the Netherlands; 4 Public Health Service region Utrecht, Zeist, the Netherlands; 5 Department of Medical Microbiology, Leiden University Medical Center, Leiden, the Netherlands; 6 Department of Viroscience, Erasmus Medical Centre, Rotterdam, the Netherlands; 7 Department of Virology, University Medical Center Utrecht, Utrecht, the Netherlands; 8 Laboratory of Medical Microbiology and Immunology, St Elisabeth Hospital, Tilburg, the Netherlands; 9 Public Health Service, Department of Infectious Diseases, Amsterdam, the Netherlands; 10 Department of Medical Microbiology, Academic Medical Center, Amsterdam, the Netherlands; 11 Department of Medical Microbiology, Division of Clinical Virology, University Medical Center Groningen, Groningen, the Netherlands; 12 http://www.rivm.nl/Onderwerpen/T/Type_Ned/Type_Ned_Virologie

**Keywords:** viral infections, viral meningitis, outbreaks, surveillance, automated surveillance, laboratory surveillance, epidemiology, laboratory, typing, VIRO-TypeNed, ECHOvirus 6, molecular surveillance, neurological symptoms

## Abstract

The Dutch virus-typing network VIRO-TypeNed reported an increase in ECHOvirus 6 (E-6) infections with neurological symptoms in the Netherlands between June and August 2016. Of the 31 cases detected from January through August 2016, 15 presented with neurological symptoms. Ten of 15 neurological cases were detected in the same province and the identified viruses were genetically related. This report is to alert medical and public health professionals of the circulation of E-6 associated with neurological symptoms.

From June 2016 onwards, an increase in the number of ECHOvirus 6 (E-6) infections was noted by the Dutch virus-typing network VIRO-TypeNed [1]. Among a total of 31 cases, 15 presented with neurological symptoms. Compared with the annual average of four cases with a neurological E-6 infection in the past five years, this increase was statistically significant. Here we aim to alert medical and public health professionals of the increase and circulation of E-6 associated with neurological symptoms.

## Epidemiological investigation

In the period from January to August 2016, 242 enterovirus (EV) cases were reported by VIRO-TypeNed [1]. E-6 was the most frequently identified type and accounted for 13% (n = 31) of the EV cases; in previous years, this type had only been detected in on average 4% of the cases, ranging from 0.3% (1/308) in 2010 to 6% (26/464) in 2015. The female:male ratio among E-6 cases was 1:1 and 18 of the 31 cases were younger than seven years (range: 2 weeks–44 years).

**Figure 1:**
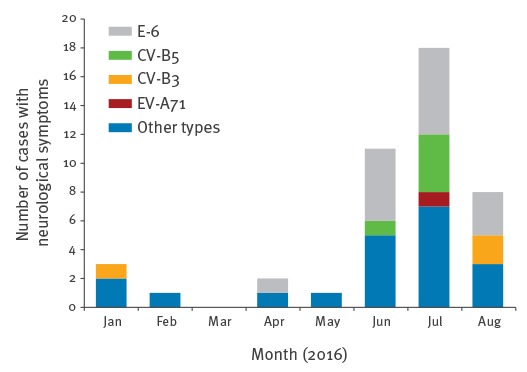
Monthly distribution of neurological cases with enterovirus infection, reported by VIRO-TypeNed, the Netherlands, January–August 2016 (n = 44)

44 (18%) of the total 242 EV cases presented with neurological symptoms and 15 of them were infected with E-6 (Figure 1, Table). Cases that presented with neurological symptoms were defined as patients with aseptic meningitis (n = 4), suspected or undefined neurological presentation (n = 2) or an (unreported) clinical presentation that prompted the physician to examine the cerebrospinal fluid (CSF) (n = 9).

E-6, CV-B3, CV-B5 and EV-A71 are shown separately as they were among the top five detected types with neurological cases. ‘Other types’ include CV-A9, CV-B1, CV-B2, CV-B4, E-5, E-7, E-9, E-13, E-18 and E-30 (Table).

**Table:** Enterovirus types reported to VIRO-TypeNed with cases presenting with neurological symptoms, the Netherlands, January–August 2016 (n = 172^a^)

EV species	EV type	Number of cases	Number of cases with neurological symptoms
EV-A	EV-A71^b^	20 (13 C2; 7 C1)	1
EV-B	CV-A9	12	3
EV-B	CV-B1	13	2
EV-B	CV-B2	6	1
EV-B	CV-B3^b^	20	3
EV-B	CV-B4	12	2
EV-B	CV-B5^b^	21	5
EV-B	E-5	3	1
EV-B	E-6^b^	31	15
EV-B	E-7	6	1
EV-B	E-9	4	1
EV-B	E-13	10	4
EV-B	E-18	2	1
EV-B	E-30	12	4

CV: Coxsackievirus; E: ECHOvirus; EV: Enterovirus.

^a^ For types CV-A2 (n = 1), CV-A5 (n = 1), CV-A6^b^ (n = 21), CV-A8 (n = 2), CV-A10 (n = 8), CV-A13 (n = 1), CV-A14 (n = 1), CV-A16 (n = 2), CV-A22 (n = 1), E-3 (n = 2), E-11 (n = 10), E-16 (n = 1), E-25 (n = 2) and EV-D68 (n = 17), no cases presenting with neurological symptoms were reported.

^b^ EV types ranked top five detected types (January to August 2016).

Case definition of neurological cases: patients with aseptic meningitis, suspected or undefined neurological presentation, encephalitis, convulsions, or with a clinical presentation (not reported) that prompted the physician to examine the cerebrospinal fluid.

The total number of cases (irrespective of EV type) presenting with neurological symptoms was not increased compared with the respective period (January through August) in the previous five years: the average in this period was 41 cases, ranging from 24 in 2013 to 50 in 2014. However, in 2016, the proportion of E-6 cases with neurological symptoms was significantly higher compared with other EV types with neurological symptoms (p value < 0.05 based on the univariable chi-squared test) (Table). Specifically, the number of cases with neurological symptoms was not increased for any of the other top five detected types (Coxsackievirus (CV)-B3, CV-B5, EV-A71 and CV-A6) (Table).

The 31 E-6 cases detected since January 2016 were identified across the Netherlands. However, from June through August 2016, when EV infections associated with neurological symptoms peaked, a cluster of 10 cases infected with the same E-6 strain were found to reside in the same province (6 female and 4 male cases, median age: 27 years; range: 2 weeks–45 years). Nine of those cases presented with neurological symptoms and included seven adults (median: 28 years; range: 27–45). Five of the 10 cases resided in the same municipality and four of them were neurological cases. These five cases included a two-week-old neonate and its mother. The child had a fever, without evident neurological symptoms. Further investigations are being conducted on the clinical and epidemiological characteristics of the cases in the cluster to investigate a possible source and link. In the same province, 36 EV cases were detected in total and clinical information was available for all; of the 26 cases infected with strains other than E-6, only six presented with neurological symptoms; they were infected with CV-B5 (n = 2), CV-B3 (n = 1), E-7 (n = 1), E-18 (n = 1) and E-30 (n = 1).

## Phylogenetic investigation 

**Figure 2:**
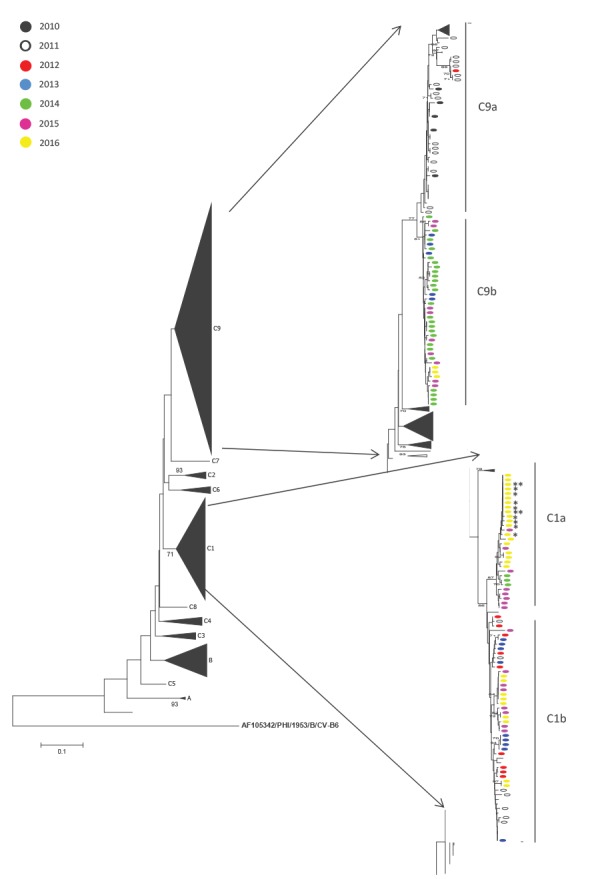
Neighbour-joining (maximum composite likelihood) tree based on a 250 nt VP1 fragment [2] of ECHOvirus 6 from patients, the Netherlands, 2010–2016 (n = 31)

In the phylogenetic analysis based on the partial VP1 region [2], the E-6 strains circulating in the Netherlands since 2010 could be grouped in the previously assigned genogroups B (one strain from 2015), C1 (75 strains from 2011–16), C4 (two strains from 2014) and C9 (65 strains from 2010–16) [3,4]. Twenty-eight of the 31 E-6 strains recovered in 2016, including those from the provincial cluster, could be characterised as C1 (Figure 2), and the three remaining strains as C9. The strains from the cluster were 99.9% homologous.

At the nodes, the percentage bootstrap support for branching events after 1,000 replicates are shown. Sequences were aligned using Simmonics sequence editor and the tree was constructed using MEGA6. The years of isolation are depicted in colour-coded circles. The 10 cases from the provincial cluster are indicated by an asterisk and the mother and child by a double asterisk.

## Outbreak detection and response

EV infection is not notifiable in the Netherlands, but more than 300 EV per year are typed in the poliovirus exclusion and EV surveillance programme. Here, we detected the outbreak in a timely manner through our regular analysis of data in the VIRO-TypeNed database, observing 10 cases in one province that clustered in time and place and had identical molecular types. The cluster was reported to the national early warning committee and surveillance unit of the national institute for public health and the environment (RIVM) in order to alert and create awareness among medical and public health professionals.

## Background

**Figure 3:**
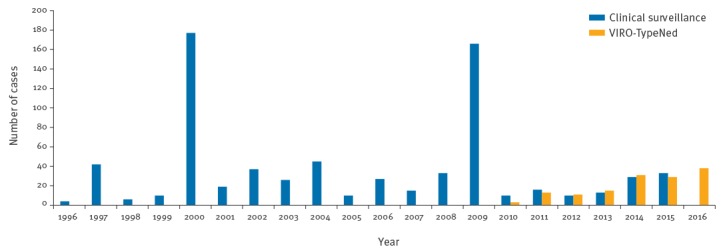
Distribution of cases of ECHOvirus 6 infection, the Netherlands, 1996–2015 (clinical surveillance; n = 728) and 2010–2016 (VIRO-TypeNed; n = 133)

E-6 is one of the five most frequently detected EV types associated with neurological symptoms worldwide, next to EV-A71, E-11, E-30 and CV-B5, each accounting for 15–20% of EV types identified in a year [5-10]. EVs are ubiquitous and circulate all year round with peaks in the summer months. Neurological symptoms can be linked to various types. However, there have been years where a majority of neurological cases could be linked to a specific type ([5-10] and data not shown), as is currently seen for E-6 in 2016. E-6 is endemic in the Netherlands and was detected more frequently in 2000 and 2009 by the clinical surveillance as described by van der Sanden et al. (Figure 3) [11].

Clinical surveillance data are collected once a year, therefore data from 2016 is not available.

Typing of positive isolates from clinical surveillance is primarily performed to exclude the circulation of poliovirus [11]. The data on non-Polio EV (NPEV) types are used to monitor NPEV circulation and trends in NPEV illness. Since 2010, these data have been collected in a standardised manner through VIRO-TypeNed. VIRO-TypeNed is a virus-typing network using a joint data-sharing database for clinical and public health laboratories to provide a more complete surveillance of EV including genetic, epidemiological, patient and clinical data such as information on gastrointestinal, respiratory and neurological symptoms [1]. Details on the surveillance system (sampling method, detection and typing methods, reporting of data) have been described elsewhere [12]. In short, all EV-positive cases detected through real-time PCR that can be characterised by partial typing of the VP1 region [2] include a minimum dataset including age and sex of patient, type of sample from which the virus was detected, whether the patient was hospitalised, travel history (by country visited) and clinical symptoms in broad categories (skin, neurological, respiratory, enteric). Cases presenting with neurological symptoms are defined as having aseptic meningitis, suspected or undefined neurological presentation, encephalitis, convulsions, or from whom clinical presentation (unreported) was such as to prompt the physician to examine the CSF.

## Discussion

On 8 August, the European Centre for Disease Prevention and Control (ECDC) published a rapid risk assessment (RRA) regarding EV detections in severe neurological cases among children and adults in Europe, to reinforce vigilance for EV-associated neurological disease [13]. Several types were reported across Europe, and alerts were released on an increase in the number of EV-A71 (a new C1 variant) associated with neurological symptoms [13] as well as on an increase of EV-D68 associated with severe respiratory disease.

The E-6 increase represents the second EV signal in the Netherlands in 2016, following the EV-D68 increase seen in the summer of 2016 [13]. Both outbreaks were rapidly detected by the national VIRO-TypeNed surveillance system [1]. As this system includes epidemiological, clinical and molecular typing data, near real-time cluster detection is feasible. At the moment, outbreak detection is based on weekly analysis of the data in the VIRO-TypeNed database. As the majority of EV infections are asymptomatic and mild, not all infections are reported. Therefore, we cannot exclude that the increase is biased based on active reports of severe (neurological) cases. However, a comparison of the data with only severe (neurological) cases in previous years revealed a clear increase for E-6; A majority of these cases were defined based on unreported clinical presentation where CSF was examined. The data on specimen type (CSF) is unbiased and completeness of this dataset is more than 90%.

Phylogenetic analysis suggested that the 2016 outbreak is associated with the C1 strain. Previous reports on E-6 outbreaks were related to C9, for example a major outbreak associated with neurological symptoms in Spain in 2008 [5] and in other countries since 2000 [3]. Analysis of full-length VP1 or full-length genomes as well as serological studies are needed to further investigate the underlying genetical and immunological factors that are responsible for the possible increase in neurological virulence and/or possible increase in circulation (e.g. viral fitness, transmissibility or lack of immunity). Of interest is that while more than half of the E-6 cases seen nationwide occurred in children, most cases belonging to the cluster were older than 27 years, suggesting that severity is not related to a younger age but rather to waning immunity or lack of immunity to the C1 strain.

Preventive measures for EV outbreaks are limited to advice on more stringent cough and hand hygiene or case isolation to prevent nosocomial spread. Treatment options for (severe) EV infections are limited. Based on the humoral responsiveness of EV infection, intravenous IG (IVIG) can be given in severe cases. However, IVIG is not always effective [14]. While antiviral drugs against EV infections are under development, there is still no EV-specific treatment available [15,16]. Several studies have described the use of the capsid inhibitor pleconaril on a compassionate use basis in neonates and immunocompromised patients with severe EV infections, with variable outcome [14]. Other options are drugs marketed for other viral infections or clinical conditions that can be used off-label, however, they have never been clinically tested in EV cases and public health implications are unknown [15].
